# Attenuating Oxidative Stress by Paeonol Protected against Acetaminophen-Induced Hepatotoxicity in Mice

**DOI:** 10.1371/journal.pone.0154375

**Published:** 2016-05-04

**Authors:** Yi Ding, Qing Li, Yuan Xu, Yuning Chen, Yue Deng, Feng Zhi, Ke Qian

**Affiliations:** 1 Department of Geriatrics, Third Affiliated Hospital of Soochow University, Changzhou, China; 2 Department of Pathology, Third Affiliated Hospital of Soochow University, Changzhou, China; 3 Modern Medical Research Center, Third Affiliated Hospital of Soochow University, Changzhou, China; University of Missouri-Kansas City, UNITED STATES

## Abstract

Acetaminophen (APAP) overdose is the most frequent cause of drug-induced acute liver failure. The purpose of this study was to investigate whether paeonol protected against APAP-induced hepatotoxicity. Mice treated with paeonol (25, 50, 100 mg/kg) received 400 mg/kg acetaminophen intraperitoneally (i.p.) and hepatotoxicity was assessed. Pre-treatment with paeonol for 6 and 24 h ameliorated APAP-induced hepatic necrosis and significantly reduced the serum alanine aminotransferase (ALT) and aspartate transaminase (AST) levels in a dose-dependent manner. Post-treatment with 100 mg/kg paeonol ameliorated APAP-induced hepatic necrosis and reduced AST and ALT levels in the serum after APAP administration for 24 h. Western blot revealed that paeonol inhibited APAP-induced phosphorylated JNK protein expression but not p38 and Erk1/2. Moreover, paeonol showed anti-oxidant activities with reducing hepatic MDA contents and increasing hepatic SOD, GSH-PX and GSH levels. Paeonol dose-dependently prevented against H_2_O_2_ or APAP-induced LDH releasing and ROS production in primary mouse hepatocytes. In addition, the mRNA levels of pro-inflammatory genes such as TNF-α, MCP-1, IL-1β and IL-6 in the liver were dose-dependently reduced by paeonol pre-treatment. Pre-treatment with paeonol significantly inhibited IKKα/β, IκBα and p65 phosphorylation which contributed to ameliorating APAP-induced hepatic inflammation. Collectively, the present study demonstrates paeonol has a protective ability against APAP-induced hepatotoxicity and might be an effective candidate compound against drug-induced acute liver failure.

## Introduction

Acetaminophen (APAP) is a safe and effective analgesic at therapeutic doses. However, APAP received at an excessively high dose can cause severe liver injury and even acute liver failure (ALF), exceeding all other causes of ALF in the United States, accounting for 46% of all cases [1]. The main mechanism of APAP-induced hepatotoxicity is due to the formation of a reactive metabolite N-acetyl-p-amino-benzoquinone imine (NAPQI), which in turn depletes glutathione (GSH) and binds to a variety of cellular mitochondrial proteins. Mitochondrial GSH depletion and covalent binding increase the generation of mitochondrial reactive oxygen species (ROS) that activate c-jun-N-terminal kinase (JNK), through upstream MAP kinase pathways [2]. Activated JNK binding to mitochondria leads to further enhancement of ROS generation, which is important in sustaining JNK activation and inducing the mitochondrial permeability transition (MPT) to mediate hepatocyte necrosis [3].

Currently, the best therapeutic option to prevent progression to liver failure of overdose patient is administration of N-acetylcysteine (NAC). When given within a relatively short time window (not later than 12 hours), NAC is effective to minimizes morbidity and mortality after APAP intoxication [4]. However, Clinical studies revealed that administration of NAC is associated with untoward side effects [5, 6]. An increasing experimental animal model revealed that the severe hepatocellular injury can lead to an intracellular, mitochondria-derived oxidant stress. After APAP overdose, ROS generated from mitochondria and other sources activate many kinases such as c-Jun N-terminal protein kinases (JNK) [7]. JNK can phosphorylate transcription factors (e.g. c-Jun, NF-κB, and ATF-2) as well as members of the Bcl-2 family to regulate apoptosis [8].

During APAP-induced hepatotoxicity, extensive cell necrosis causes the release of cell contents such as nuclear DNA fragments, mitochondrial DNA (mtDNA), heat shock proteins, hyaluronic acid. It is well recognized that these cellular components as damage-associated molecular patterns (DAMPs) can bind to various pattern recognition receptors to induce pro-inflammatory cytokines production. Furthermore, extensive cytokines and chemokines recruit neutrophils and monocytes into the liver with the potential to aggravate the initial injury [9]. In addition, resident Kupffer cells and accumulating macrophages contribute to the overall liver injury during APAP-induced hepatotoxicity. It has been reported that pretreatment of mice with macrophage inactivators decreases acetaminophen hepatotoxicity and the formation of ROS and reactive nitrogen species (RNS) [10].

Paeonol (2’-hydroxy-4’-methoxyacetophenone, C_9_H_10_O_3_) is one of the main active compounds in Moutan Cortex root, which has been widely in traditional Chinese medicine. It has been reported that paeonol possesses an anti-inflammation property such as attenuating airway inflammation and hyperresponsiveness in a murine model of ovalbumin-induced asthma [11], cigarette smoke-induced lung inflammation by inhibiting ROS-sensitive inflammatory signaling [12] and protecting rat vascular endothelial cells from ox-LDL-induced injury via downregulating microRNA-21 expression and TNF-α release [13]. In addition, accumulating evidence indicates that paeonol exhibits anti-inflammatory and free-radical scavenging properties to prevent against neurodegeneration and neurotoxicity. Tseng YT et al. reported that microglia-mediated inflammation and oxidative stress-induced neurotoxicity was attenuated by paeonol in rat primary microglia and cortical neurons [14]. Liu et al. reported that peaonol ameliorated cognition deficits of diabetic encephalopathy in streptozotocin-induced diabetic rat [15]. Paeonol also reduced cerebral infarction involving the superoxide anion and microglia activation in ischemia-reperfusion injured rats [16]. However, few study illustrates the ability of paeonol in drug-induced hepatotoxicity. In the present study, we evaluated the effects of paeonol on APAP-induced hepatotoxicity in mice. Here, we reported that paeonol exhibited remarkable dose-dependent hepatoprotection against APAP-induced injury, potentially through ameliorating oxidative stress.

## Materials and Methods

### Material

Acetaminophen, paeonol, sodium carboxymethyl cellulose, N-Acetyl-L-cysteine, hematoxylin and eosin were purchased from Sigma Aldrich (St. Louis, MO, USA). NF-κB pathway antibody sampler kit (#9936), MAPK family antibody sampler kit (#9926), Phospho-MAPK family antibody sampler kit (#9910) and anti-β-actin antibody (#4970) were purchased from Cell Signaling Technology (Beverly, MA, USA). Trizol reagent and SYBR^®^ Green PCR Master Mix were purchased from Invitrogen (Carlsbad, CA, USA). 2,7-dichlorofluorescein diacetate (DCFH-DA) purchased from Life Technologies (Grand Island, NY).

### Animals and treatment

Male C57BL/6 mice, 6–8 weeks of age, were purchased from Experimental Animal Center of Yangzhou University (Jiangsu, China). They were maintained with free access to pellet food and water in stainless-steel cages at a temperature of 21± 2°C with a 12 h light/dark cycle. All the animals received humane care in compliance with Guide for the Care and Use of Laboratory Animals. All the animal experiments were approved by Soochow University Animal Care and Use Committee (SDU-ACUC) and were arranged to minimize suffering and to reduce the number of animals used. Paeonol was suspended in 0.5% carboxymethylcellulose sodium (CMC-Na) solution to a final concentration of 50 mg/ml. For paeonol pre-treatment, mice were administered with vehicle, 25, 50, 100 mg/kg paeonol by gavage once daily for three days or NAC (300 mg/kg) by intraperitoneal (i.p.) injection at day 3 according to previous study [15, 17]. To induce APAP hepatotoxicity, mice were injected intraperitoneally with 400 mg/kg APAP for 6 h and 24 h at day 3. For paeonol post-treatment, after intraperitoneal injection with 400 mg/kg APAP for 30 min, mice were administered with vehicle, 25, 50, 100 mg/kg paeonol by gavage for 24 h. All mice were anesthetized with isoflurane and sacrificed for the collection of blood and liver samples.

### Histological analysis

For evaluation of hepatotoxicity, fresh liver tissue samples were kept in 10% formalin solution, and paraffin blocks were prepared subsequently. Paraffin-embedded liver sections were stained with hematoxylin-eosin (H&E). The images were visualized by microscopy (IX73, Olympus, Japan).

### Determination of plasma ALT and AST concentrations

Aspartate aminotransferase (AST), alanine aminotransferase (ALT) were quantified in serum using a commercial kit. (Nanjing Jiancheng bioengineering institute, Nanjing, China).

### Measurement of SOD, MDA, GSH-PX and GSH in liver tissues

Hepatic homogenates were used for the determination of SOD, MDA, GSH-PX and GSH levels by using a commercial kit (Nanjing Jiancheng bioengineering institute, Nanjing, China). The results were corrected for their protein content.

#### Isolation of primary mouse hepatocytes and LDH leakage assay

Briefly, primary mouse hepatocytes were isolated from C57BL/6J mice by perfusion with collagenase type IV. After perfusion, hepatocytes were purified at 50 g centrifugation and cultured in DMEM medium (Invitrogen Corp., Carlsbad, CA) containing 10% fetal bovine serum (GIBCO, Grand Island, NY) and antibiotics (100 U/ml penicillin and 100 U/ml streptomycin) in a humidified atmosphere of 5% CO_2_ at 37°C. For lactate dehydrogenase (LDH) leakage assay, hepatocytes were cultured in collagen-coated 48-well plate and treated with paeonol at different concentrations in the absence or presence of 5 mM APAP or 250 μM H_2_O_2_ for 6 h. LDH in the culture medium and whole-cell lysate was determined using a commercial kit (Nanjing Jiancheng bioengineering institute, Nanjing, China), and released LDH percentage was calculated using the following formula of LDH media/ (LDH media + LDH whole-cell lysate) × 100%.

### ROS measurements

Briefly, hepatocytes were cultured in collagen-coated 96-well black plates with transparent bottoms and treated with paeonol at different concentrations in absence or presence of 5 mM APAP or 250 μM H_2_O_2_ for 6 h. The hepatocytes were subsequently loaded with 10 μM DCFH-DA for 30 min at 37°C. After washing twice with PBS. Fluorescence intensity in cells was detected at excitation and emission wavelengths of 490 nm and 520 nm using a fluorescence microplate reader (Molecular Devices, Sunnyvale, CA).

### Real-time PCR

Total RNA from liver was isolated using Trizol Reagent (Invitrogen, Carlsbad, CA) according to the manufacturer’s instructions. The extracted RNA (2 μg) was used for reverse transcription with M-MLV reverse transcriptase (Life Technologies, Grand Island, NY). RT-PCR was performed with the ABI Prism 7000 sequence detection system (Applied Biosystems, Foster City, CA) using SYBR^®^ Green PCR Master Mix (Life Technologies, Grand Island, NY). Each sample was run and analyzed in duplicate. Specific RT-PCR primers were listed in Table 1. Target mRNA levels were adjusted as the values relative to GAPDH, which was used as the endogenous control.

**Table 1:** Primers used for real-time quantitative PCR.

Name	Sequence (5’-3’)
IL-1β	Sense: TCCAGGATGAGGACATGAGCAC
	Antisense: GAACGTCACACACCAGCAGGTTA
IL-6	Sense: CCACTTCACAAGTCGGAGGCTTA
	Antisense: GCAAGTGCATCATCGTTGTTCATAC
TNF-α	Sense: CACGTCGTAGCAAACCACCAA
	Antisense: CCCATTCCCTTCACAGAGCAA
MCP-1	Sense: CAGGTCCCTGTCATGCTTCT
	Antisense: TCTGGACCCATTCCTTCTTG
GAPDH	Sense: GTCGTGGATCTGACGTGCC
	Antisense: TGCCTGCTTCACCACCTTCT

### Western blot

Briefly, liver tissues were homogenized in RIPA buffer (Cell Signaling Technology, Danvers, MA). Equal amount of protein were separated by 10% SDS-PAGE and electro-transferred onto a polyvinylidene diuoride membrane (Millipore Corp., Bedford, MA). The membrane was blocked with 5% nonfat milk and incubated with primary antibodies overnight at 4°C. The membranes were blotted with horseradish peroxidase (HRP)-conjugated secondary antibody for 1 h. Protein bands were visualized using western blotting detection system according to the manufacturer’s instructions. The densities of protein bands were determined by Image J software.

### Statistical analysis

All data were represented as means ± S.E.M of three independent experiments. All statistical analyses were conducted using SPSS software. One way ANOVA was used for statistical analyses of the data. *P* < 0.05 was considered to be statistically significant.

## Results

### Paeonol ameliorated APAP-induced hepatic necrosis

**Fig 1:**
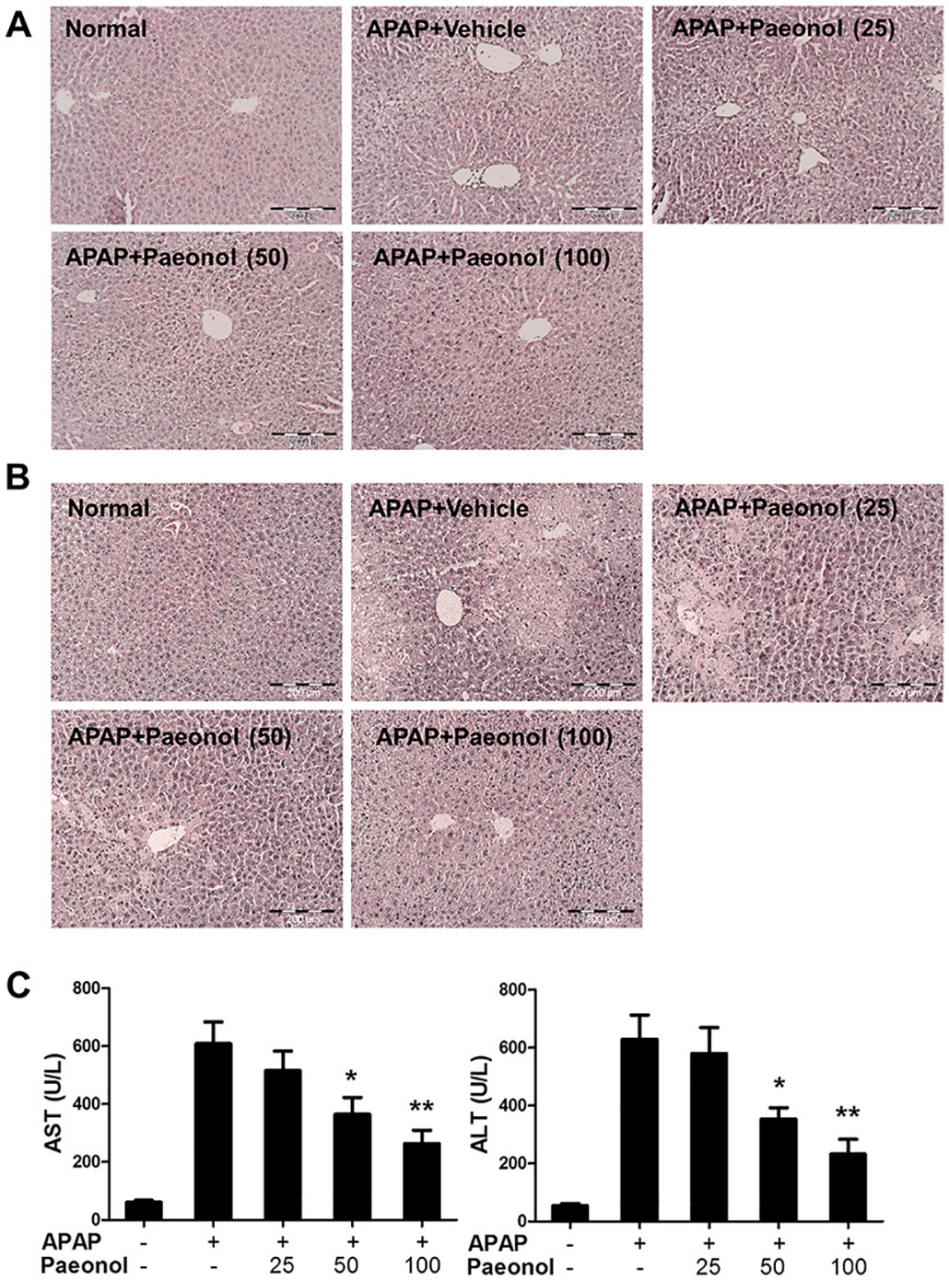
Pre-treatment with paeonol protected against APAP-induced liver injury. Mice were administered with either vehicle (0.5% CMC-Na) or paeonol (25, 50, 100 mg/kg) by gavage for three days. At day 3, mice were intraperitoneally (i.p.) injected with either 400 mg/kg APAP or an equal volume of PBS. Liver tissues were collected after APAP administration for 6 h (A) and 24 h (B). Liver sections were stained with H&E. Magnification: 200X. (C) After APAP administration for 6 h, blood was collected and the levels of ALT and AST in the serum were determined by a commercially available kit. Data are shown as means ± S.E.M. *P<0.05, **P<0.01 v.s. APAP treatment (n = 8).

To determine the preventive effects of paeonol on APAP-induced hepatotoxicity, mice were pre-treated with different doses of paeonol by gavage once daily for three days. To induce hepatotoxicity, APAP (400 mg/kg) was administered by intraperitoneal (i.p.) injection at day 3. H&E staining results showed that APAP administration caused massive necrosis in the liver. Pre-treatment with paeonol (50 and 100 mg/kg) ameliorated APAP-induced hepatic necrosis at 6 and 24 h (Fig 1A and 1B). AST and ALT, the markers of liver injury, were dramatically increased after APAP treatment for 6 h. Consistent with histological analysis data, pre-treatment with paeonol significantly inhibited the serum AST and ALT levels in APAP-treated mice in a dose-dependent manner (Fig 1C).

**Fig 2:**
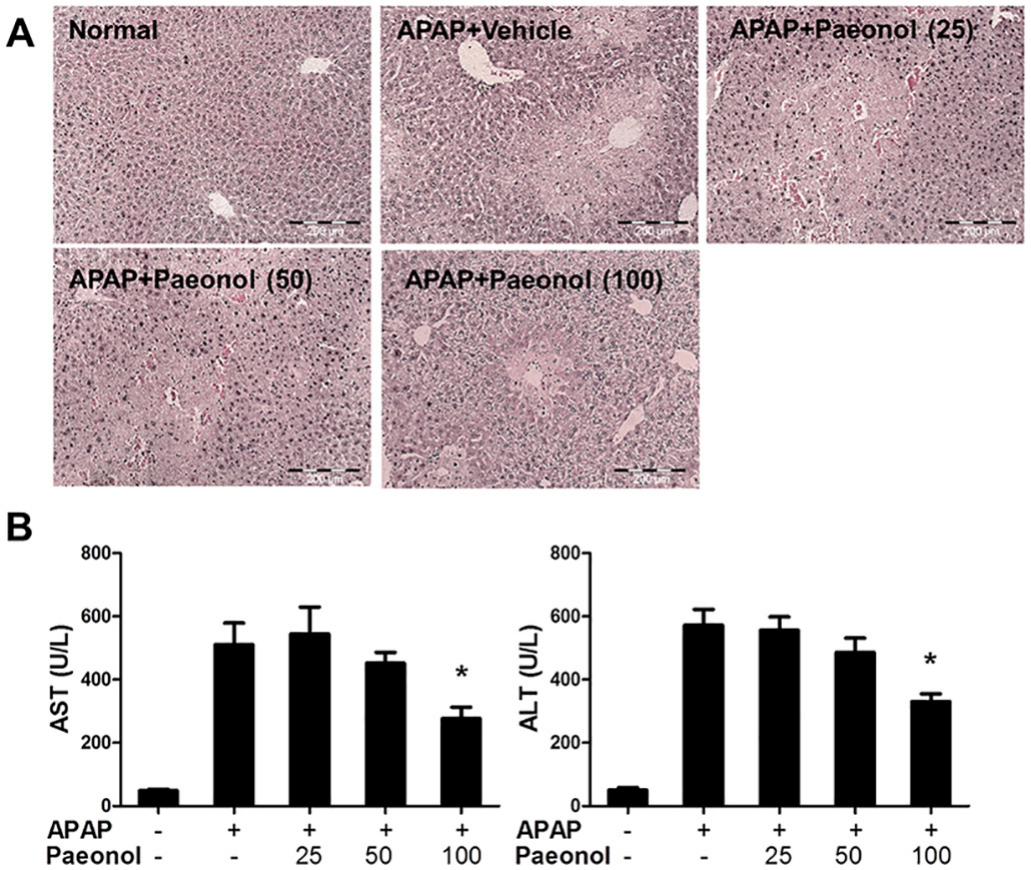
Post-treatment with paeonol protected against APAP-induced liver injury. Mice were intraperitoneally injected with either 400 mg/kg APAP or an equal volume of PBS. After 30 min, mice were administered with either vehicle (0.5% CMC-Na) or paeonol (25, 50, 100 mg/kg) by gavage. Liver tissue and blood were collected after paeonol treatment for 24 h. (A) Liver sections were stained with H&E. Magnification: 200X. (B) The levels of ALT and AST in the serum were determined by a commercially available kit. Data are shown as means ± S.E.M. *P<0.05, **P<0.01 v.s. APAP treatment (n = 8).

Next, we evaluated the curative effects of paeonol on APAP-induced hepatotoxicity. After APAP administration for 30 mins, mice were treated with different doses of paeonol by gavage for 24 h. Histological analysis showed that post-treatment with 100 mg/kg paeonol ameliorated APAP-induced hepatic necrosis and reduced AST and ALT levels in the serum (Fig 2A and 2B). These data indicated that paeonol protected against APAP-induced hepatotoxicity.

### The effect of Paeonol on APAP-induced MAPK pathway activation in the liver

**Fig 3:**
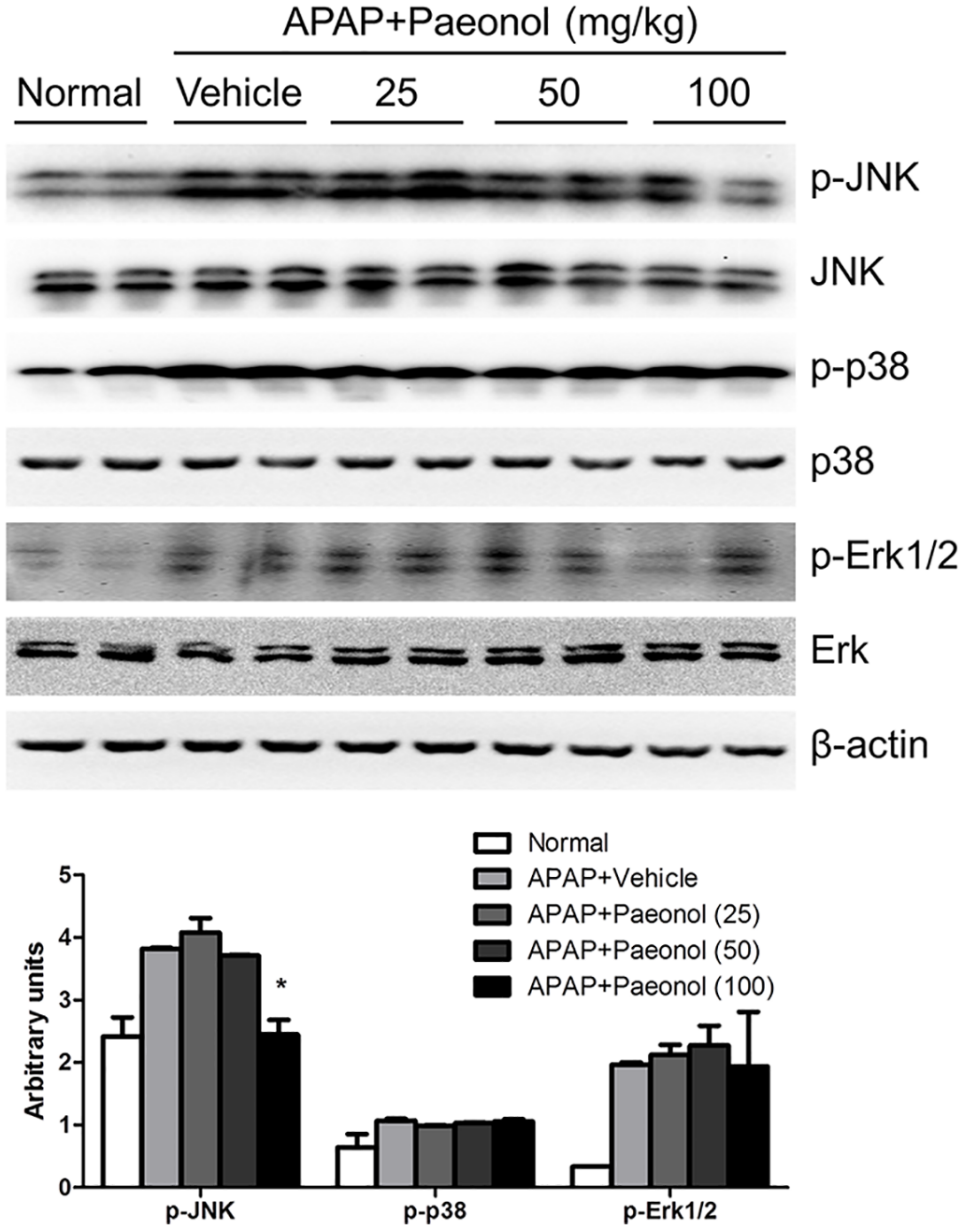
Paeonol inhibited APAP-induced MAPK pathway activation. The protein levels of total and phosphorylated p38, JNK and Erk1/2 in the liver were determined by western blot. β-actin was used as the endogenous control. The representative data are shown and bands were analyzed by densitometry. Data are shown as means ± S.E.M. *P<0.05, **P<0.01 v.s. APAP treatment.

It is generally accepted that APAP-induced hepatotoxicity leads to mitochondrial oxidant stress, which triggers the activation of several MAP kinases [18]. Therefore, we evaluated the effect of paeonol on MAPK pathway activation in APAP-treated liver. As shown in Fig 3, APAP induced p38, Erk1/2 and JNK phosphorylation in the liver. Interestingly, paeonol selectively inhibited JNK phosphorylation but not p38 and Erk1/2, suggesting that JNK kinase was involved in protective role of paeonol against APAP-induced hepatotoxicity.

### The effects of Paeonol on APAP-induced oxidative stress in mice

**Fig 4:**
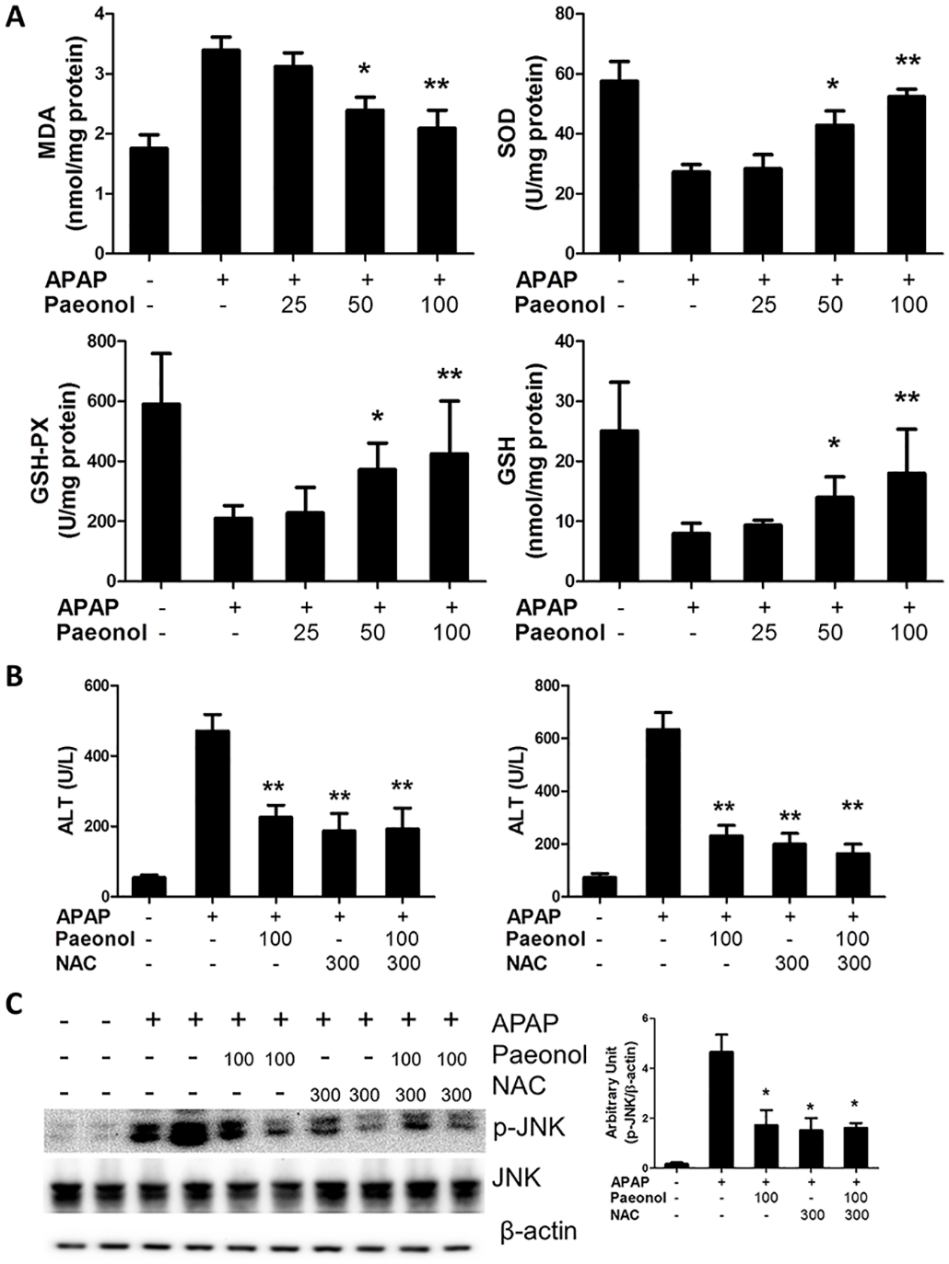
Paeonol reduced APAP-induced hepatic oxidative stress. (A) Mice were administered with either vehicle (0.5% CMC-Na) or paeonol (25, 50, 100 mg/kg) by gavage for three days. At day 3, mice were intraperitoneally injected with either 400 mg/kg APAP or an equal volume of PBS. Liver was collected and hepatic homogenates were used for the determination of MDA, SOD, GSH and GSH-PX levels by using commercial kits. (B) Mice were administered with either vehicle (0.5% CMC-Na) or paeonol (25, 50, 100 mg/kg) by gavage for three days. At day 3, mice were co-treated with either 400 mg/kg APAP and 300 mg/kg NAC by intraperitoneal injection. The levels of ALT and AST in the serum were determined by a commercially available kit. (C) The protein levels of total and phosphorylated JNK in the liver were determined by western blot. β-actin was used as the endogenous control. The representative data are shown and bands were analyzed by densitometry. Data are shown as means ± S.E.M. *P<0.05, **P<0.01 v.s. APAP treatment (n = 8).

The toxicity of APAP is initiated by cytochrome P450-mediated reactions that convert APAP to NAPQI. We evaluated the effect of paeonol on cytochrome P450 enzymes. Real-time PCR and western blot results showed pre-treatment with paeonol had no effect on Cyp1a2 and Cyp2e1 mRNA and protein levels (S1 Fig), suggesting that paeonol might not involve in APAP metabolic activation. NAPQI depletes GSH and increase ROS generation to mediate hepatocyte necrosis. Numerous studies have shown that oxidative stress plays a key role in drug-induced acute liver failure. Therefore, the effects of paeonol on oxidative stress were determined. Although paeonol pre-treatment for 30 mins had no effect on APAP—induced GSH depletion (S2 Fig), pre-treatment with paeonol significantly prevented APAP-induced MDA increasing in a dose-dependent manner after APAP administration for 6 h. Moreover, SOD recognized as an important antioxidant enzyme was up-regulated in paeonol-pretreated mice (Fig 4A). In addition, we found that pre-treatment with paeonol prevented total GSH and GSH-PX levels decreasing after APAP administration for 6 h, which indicated that paeonol had a strong antioxidant capacity against APAP-induced liver injury. As an anti-oxidant, NAC is the best therapeutic option to prevent progression to drug-induced liver failure. After pre-treatment with paeonol for 3 days, mice were co-treated with NAC and APAP for 6 h. The results showed that 100 mg/kg paeonol pre-treatment, NAC alone and NAC+paeonol treatment significantly reduced ALT and AST levels in the serum and JNK phosphorylation. However, there was no significant difference about ALT and AST levels and JNK phosphorylation between NAC+paeonol and NAC alone treatment (Fig 4B and 4C). Co-treatment with NAC and paeonol did not further improve APAP-induced hepatotoxicity compare to NAC alone treatment. These data indicate that paeonol may works as antioxidant to ameliorate APAP-induced hepatotoxicity.

### Paeonol reduced hepatic oxidative stress *in vitro*

**Fig 5:**
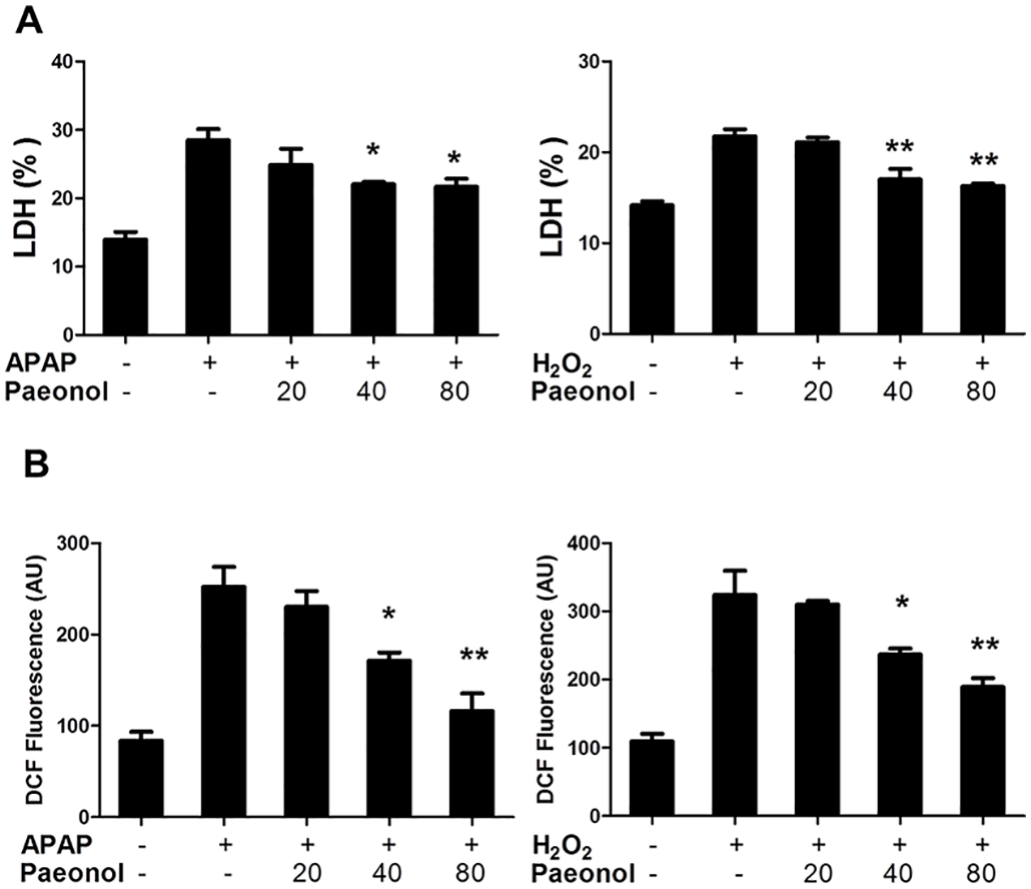
Paeonol prevented against H_2_O_2_ or APAP-induced LDH releasing and ROS production in primary mouse hepatocytes. Primary mouse hepatocytes were treated with paeonol (20, 40, 80 μM) in the absence or presence of 5 mM APAP or 250 μM H_2_O_2_ for 6 h. (A) LDH leakage percentage were determined by a commercial kit. (B) ROS formation was measured using a fluorescence microplate reader. Data are shown as means ± S.E.M. *P<0.05, **P<0.01 v.s. APAP treatment.

To confirm the effect of paeonol on oxidative stress, we isolated primary hepatocytes and performed cell-based in vitro assays. As shown in Fig 5A, Paeonol reduced LDH leakage in the supernatant in a dose-dependent manner, suggesting that paeonol protected primary hepatocytes from APAP-induced cytotoxicity. In agreement with APAP treatment, paeonol also protected primary hepatocytes from H_2_O_2_-induced cytotoxicity. We further determine ROS levels in APAP or H_2_O_2_-treated hepatocytes to confirm the effect of paeonol on anto-oxidative stress. As shown in Fig 5B, paeonol significantly reduced APAP or H_2_O_2_-induced ROS production in a dose-dependent manner in primary hepatocytes.

### Paeonol inhibited APAP-induced hepatic inflammation

**Fig 6:**
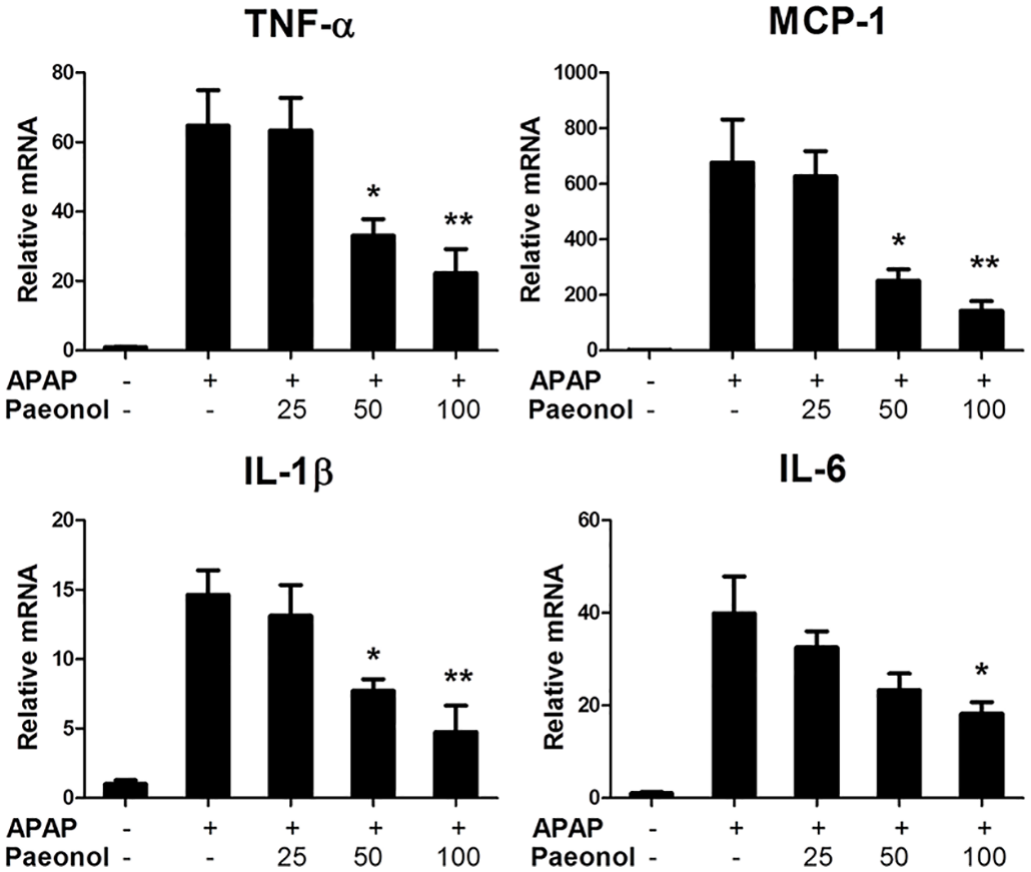
Paeonol inhibited APAP-induced hepatic inflammation. Total RNA from liver was isolated and hepatic mRNA levels of pro-inflammatory genes were determined by qPCR. GAPDH was used as the endogenous control. Data are shown as means ± S.E.M. *P <0.05, **P<0.01 v.s. APAP treatment (n = 8).

APAP-induced hepatocyte necrosis activates Kupffer cells and causes severe hepatic inflammation. To determine the effect of paeonol on hepatic inflammation induced by APAP treatment, the mRNA levels of pro-inflammatory genes were determined using real-time PCR. Mice administrated with APAP had severe hepatic inflammation with a significant increasing of TNF-α, MCP-1, IL-1β and IL-6 mRNA expression compared to control mice. Paeonol pre-treatment markedly reduced these genes mRNA expression in a dose-dependent manner (Fig 6).

### Paeonol suppressed NF-κB pathway activation in APAP-treated liver

**Fig 7:**
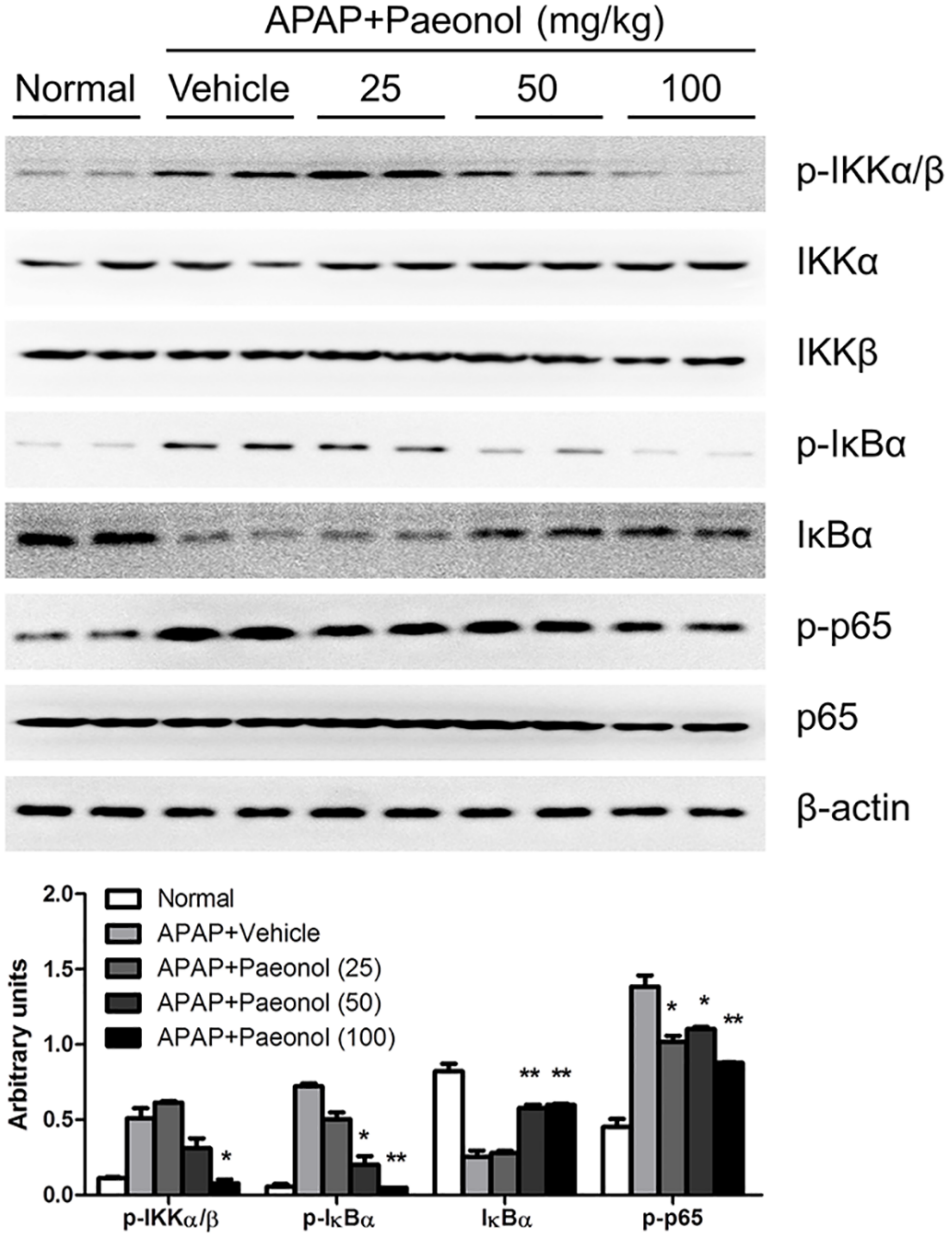
Paeonol significantly inhibited IKKα/β, IκBα and p65 phosphorylation in APAP-treated liver. The protein levels of total and phosphorylated IKKα/β, IκBα and p65 in the liver were determined by western blot. β-actin was used as the endogenous control. The representative data are shown and bands were analyzed by densitometry. Data are shown as means ± S.E.M. *P<0.05, **P<0.01 v.s. APAP treatment.

Transcription factor such as NF-kB is believed to play an important role in regulating the expression of genes that involved in tissue damage and inflammation [19]. Western blotting results indicated that APAP significantly induced IKKα/β, IκBα and p65 phosphorylation, which contributes to regulation of TNF-α, MCP-1, IL-1β and IL-6 mRNA expression. Peaonol pre-treatment dramatically reduced IKKα/β, IκBα and p65 phosphorylation in APAP-treated liver (Fig 7), suggesting that peaonol protected against APAP-induced hepatic inflammation through inhibition of NF-κB pathway activation.

## Discussion

Severe APAP hepatotoxicity frequently leads to acute liver failure. APAP is safe and widely used as an analgesic and antipyretic drug. However, unintentional overdosing is usually only recognized after symptoms have developed [20]. Since NAC was developed as the first clinical antidote for APAP hepatotoxicity, no further therapeutic intervention has been developed during the last 40 years [21]. Although NAC effectively prevents APAP-induced hepatotoxicity and avoid greater morbidity and mortality, some patients still develop toxicity presenting with complicated overdose scenarios [22]. Therefore, therapeutic options and approaches for drug-induced hepatotoxicity need to be developed. In this study, we described the effect of paeonol on APAP-induced hepatotoxicity. Our data indicated that paeonol prevented against APAP-induced hepatocytes necrosis with the serum ALT and AST levels decreasing. It was known that APAP-induced hepatotoxicity is due to the formation of a reactive metabolite NAPQI via the cytochrome P-450 pathway, which in turn depletes glutathione. Although we found that paeonol may not involve in APAP metabolic activation, peaonol significantly inhibited APAP-induced oxidative stress. Several studies have demonstrated that paeonol is a safe agent that has a protective effect against injury in different organs such as cerebral ischemic injury [23], DSS-induced murine colitis [24] and cigarette smoke-induced lung inflammation [11]. As a natural product, peaonol processes a broad range of clinically curative effect. Nowadays, the search for new drugs and novel therapeutic intervention strategies increasingly includes testing plant extracts and other natural products. It is generally accepted that natural products have multiple functions such as anti-oxidative, anti-inflammation, anti-virus, anti-cancer. Therefore, there is a critical and urgent need to explore the therapeutic potential of natural products and other compounds for prevention and treatment of hepatotoxicity. As an experimentally convenient and clinically relevant animal model, Acetaminophen-induced hepatotoxicity is attractive and widely used to evaluate therapeutic potential of drug candidates [25].

Oxidative stress is a mechanism that has been postulated to be important in the development of APAP hepatotoxicity. The massive impairment of antioxidant defense systems was involved in APAP-induced hepatotoxicity [26]. In the present study, we found that peaonol reduced hepatic MDA contents in APAP-treated mice, which is a naturally occurring product of lipid peroxidation. On the contrary, hepatic SOD, GSH-PX and GSH levels were increased by paeonol treatment (Fig 4). In addition, paeonol prevented against H_2_O_2_ or APAP-induced ROS production in primary mouse hepatocytes (Fig 5). Moreover, Co-treatment with NAC and paeonol did not further reduce APAP-induced AST and ALT levels in the serum and JNK phosphorylation compared to NAC alone treatment. These data suggested that paeonol may exhibit the similar function as NAC to protect against APAP-induced hepatotoxicity through ameliorating impairment of antioxidant defense systems. ROS production was regulated by many factors such as nuclear factor-like (Nrf) 2 pathway [27], NADPH oxidase [28] and mitochondria [29]. It has been reported that NADPH oxidase-dependent ROS generation was involved in the effect of paeonol on decreasing pro-inflammatory cytokines production in THP-1 macrophages [30]. Zhao et al reported that paeonol upregulated Nrf2 expression during cerebral ischemic injury [23]. Zhou et al reported that paeonol treatment upregulated cytochrome oxidase levels in hippocampal and cortical tissues, which may be related to increased mitochondrial function [31]. However, the underlying mechanism of paeonol to regulate antioxidant defense systems in hepatotoxicity remains unknown.

It is well known that drug toxicity caused hepatocytes necrosis, which results in neutrophils and monocytes infiltration. However, it is controversial whether neutrophils and macrophages actually enhance the injury or contribute to the repair and recovery of the damaged liver by removing cell debris [32]. It has been reported that deficiencies in the expression of IL-10 in mice may increase susceptibility to APAP-induced hepatotoxicity by enhancing the levels of pro-inflammatory cytokines and nitric oxide synthase within the liver [33]. Consistent with the previous studies, our study showed that APAP injection induced significantly pro-inflammatory cytokines production in the liver. Peaonol treatment inhibited these pro-inflammatory genes expression in the liver (Fig 6). NF-κB has served as an important transcription factor for inducible transcription of inflammatory cytokine expression [34]. Western blot assay from our study showed that paeonol treatment inhibited APAP-induced IKKα/β phosphorylation, which, in turn, inhibited NF-κB activation (Fig 7). In addition, peaonol treatment also inhibited JNK phosphorylation but not Erk1/2 and p38 (Fig 3), which is an important component of stress response in APAP-induced hepatotoxicity [35]. Many studies reported that ROS production activated NF-κB and MAPK pathway [35, 36]. In addition, JNK, as a serine/threonine kinase, is important in responding to cytokines production. The JNK-dependent pathway is involved in TNF-α-induced NF-κB activation [37]. However, it remains unclear if paeonol directly target NF-kB activation and cytokine formation after APAP treatment. The relationship among decreasing ROS production, NF-κB and JNK activation induced by peaonol was still poorly understood.

In summary, the results from our study revealed that paeonol treatment has a great positive effect on attenuating oxidative stress and inflammation in APAP-induced hepatotoxicity. Many natural medicines provide a superior therapeutic effect via modulation of balance of multiple targets, and results in decreased side effect profile compared to modulation of a single target by a single selective ligand. However, additional work will be required to determine the underlying mechanism involved in the effect of paeonol on hepatotoxicity. A deep-going study on the biologic activity of paeonol will help in developing natural medicines-based therapeutic interventions for drug-induced acute liver failure.

## Supporting Information

S1 Fig The effect of paeonol on cytochrome P450 enzymes. Mice were administered with vehicle, 25, 50, 100 mg/kg paeonol by gavage once daily for three days. To induce APAP hepatotoxicity, mice were injected intraperitoneally with 400 mg/kg APAP for 30 mins at day 3. (A) Total RNA from liver was isolated and hepatic mRNA levels of Cyp1a2 and Cyp2e1 were determined by qPCR. GAPDH was used as the endogenous control. (B) The protein levels of Cyp1a2 and Cyp2e1 in the liver were determined by western blot. β-actin was used as the endogenous control. The representative data are shown and bands were analyzed by densitometry. Data are shown as means ± S.E.M. *P <0.05, **P<0.01 v.s. APAP treatment (n = 8).
(TIF)

S2 Fig The effect of paeonol on hepatic GSH level. Mice were administered with vehicle, 25, 50, 100 mg/kg paeonol by gavage once daily for three days. To induce APAP hepatotoxicity, mice were injected intraperitoneally with 400 mg/kg APAP for 30 mins at day 3. Liver tissues were collected and hepatic homogenates were used for the determination of GSH levels by using commercial kits. Data are shown as means ± S.E.M. *P<0.05, **P<0.01 v.s. APAP treatment (n = 8).
(TIF)
