# Supplementary figures and images for: Attenuating Oxidative Stress by Paeonol Protected against Acetaminophen-Induced Hepatotoxicity in Mice
Source: PLoS One. 2016 May 4;11(5):e0154375. doi: 10.1371/journal.pone.0154375 (PMC4856301; doi:10.1371/journal.pone.0154375)

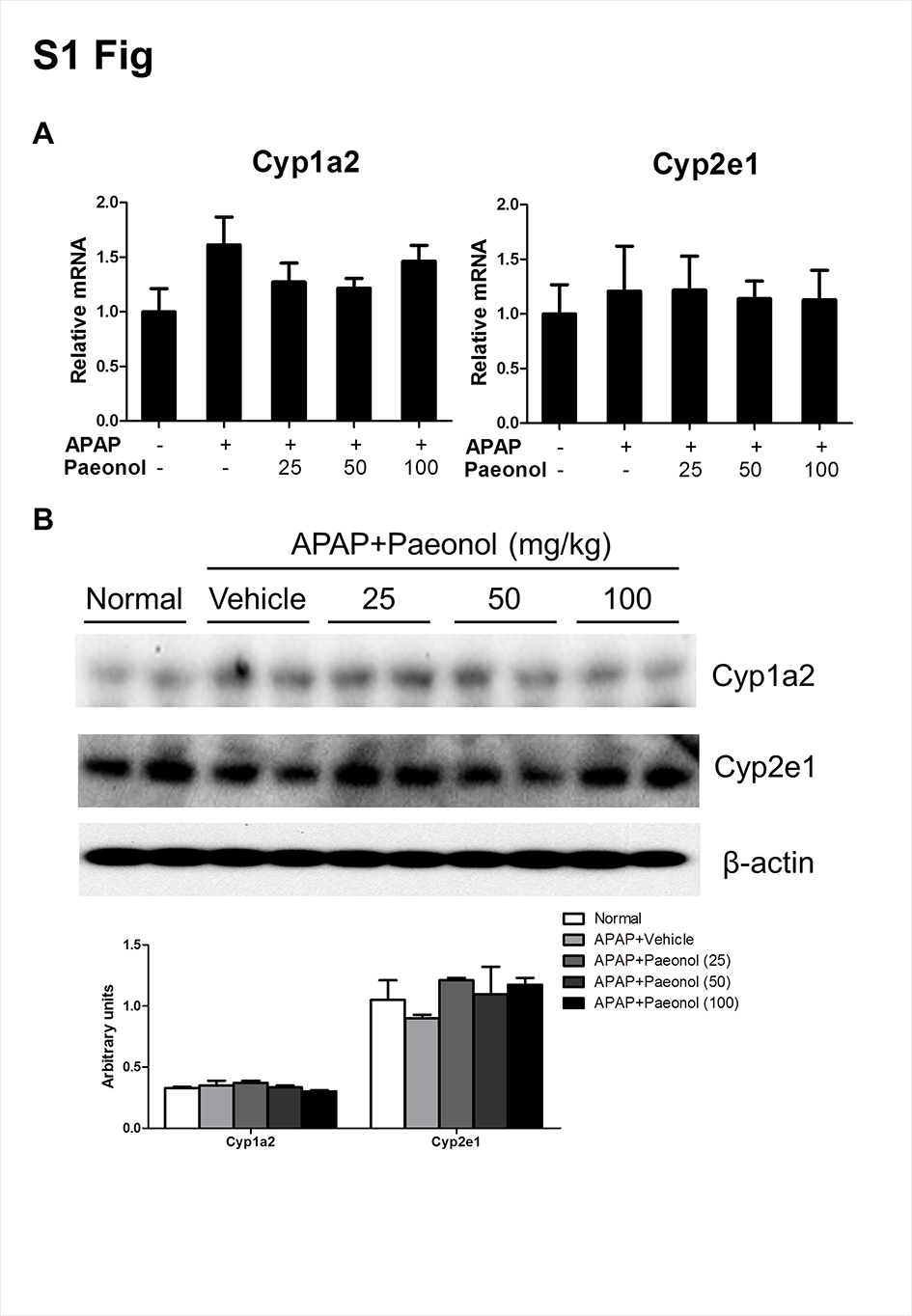

Supplement: S1 Fig — Mice were administered with vehicle, 25, 50, 100 mg/kg paeonol by gavage once daily for three days. To induce APAP hepatotoxicity, mice were injected intraperitoneally with 400 mg/kg APAP for 30 mins at day 3. (A) Total RNA from liver was isolated and hepatic mRNA levels of Cyp1a2 and Cyp2e1 were determined by qPCR. GAPDH was used as the endogenous control. (B) The protein levels of Cyp1a2 and Cyp2e1 in the liver were determined by western blot. β-actin was used as the endogenous control. The representative data are shown and bands were analyzed by densitometry. Data are shown as means ± S.E.M. *P <0.05, **P<0.01 v.s. APAP treatment (n = 8). (TIF) [file pone.0154375.s001.TIF]

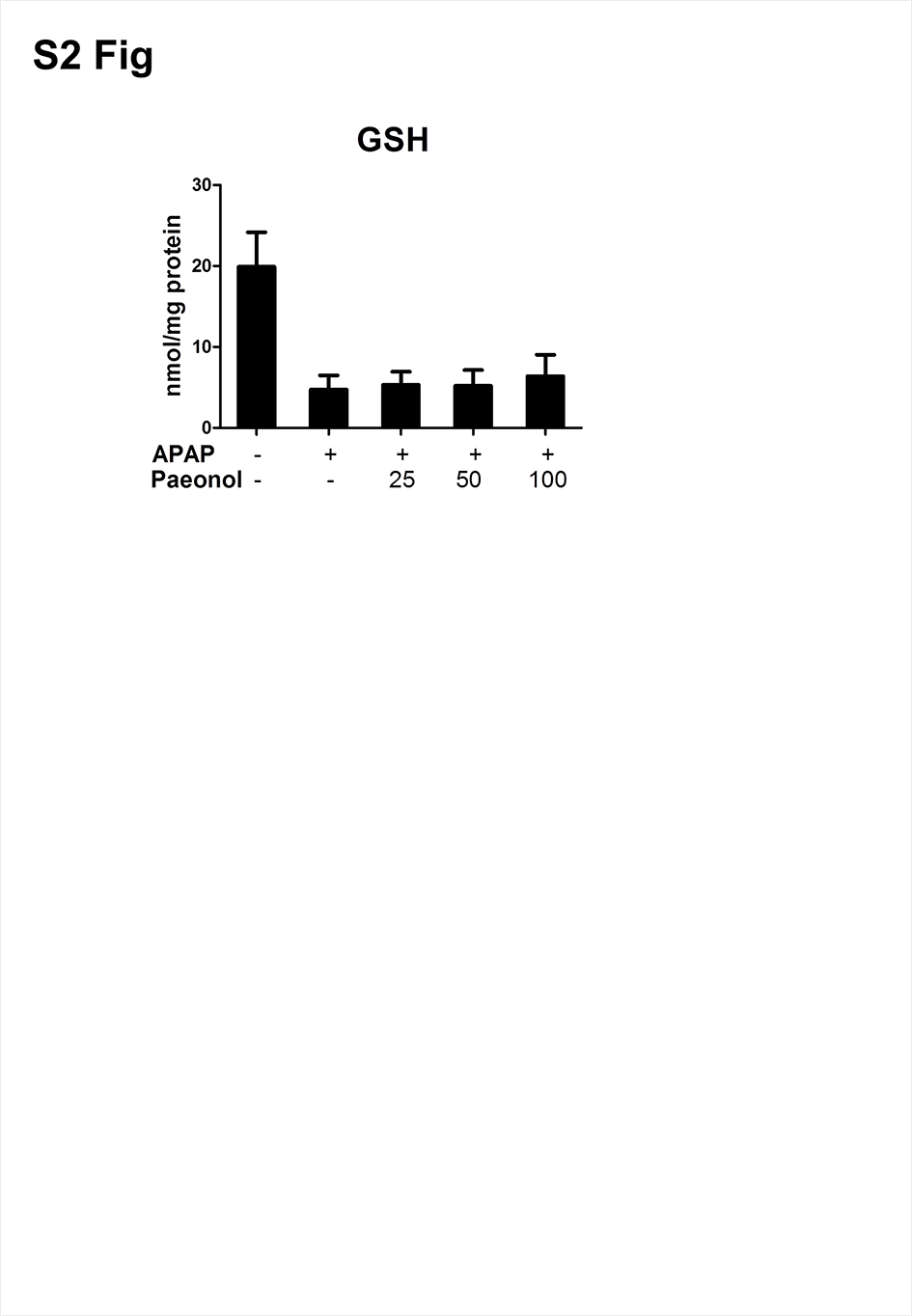

Supplement: S2 Fig — Mice were administered with vehicle, 25, 50, 100 mg/kg paeonol by gavage once daily for three days. To induce APAP hepatotoxicity, mice were injected intraperitoneally with 400 mg/kg APAP for 30 mins at day 3. Liver tissues were collected and hepatic homogenates were used for the determination of GSH levels by using commercial kits. Data are shown as means ± S.E.M. *P<0.05, **P<0.01 v.s. APAP treatment (n = 8). (TIF) [file pone.0154375.s002.TIF]
